# Binding of Antimicrobial Peptide Indolicidin to DMPC Bilayer Using Replica-Exchange Molecular Dynamics

**DOI:** 10.1021/acs.jcim.5c01153

**Published:** 2025-08-21

**Authors:** Alex R. Fitz, Dmitri K. Klimov, Christopher Lockhart

**Affiliations:** School of Systems Biology, 3298 George Mason University, Manassas, Virginia 20110, United States

## Abstract

Cationic antimicrobial peptides (AMPs) are toxic to microbes, such as bacteria and fungi, and have been increasingly studied as an alternative to traditional antibiotics, in part because AMPs are bactericidal with a minimum risk of developing bacterial resistance. Indolicidin (IL) is an AMP derived from bovine neutrophils that is unique due to its high prevalence of tryptophan and proline amino acids and its disordered structure. In addition to its antimicrobial activity, IL has exhibited toxicity toward mammalian cells, resulting in hemolysis. Although the precise physicochemical mechanism of IL cytotoxicity is unknown, its interactions with lipid bilayers are the primary focus of investigation. We conducted all-atom replica-exchange molecular dynamics simulations with solute tempering (REST) to rigorously explore the interactions between IL and a dimyristoylphosphatidylcholine (DMPC) bilayer and establish the atomistic basis of IL binding. We also performed REST simulations of IL in water to probe the conformational changes in IL between water and bilayer environments. Our simulations demonstrate that IL, which predominantly adopts random coil conformations in both environments, loses turn structure and tertiary contacts, extending upon binding to the bilayer. IL interactions with the bilayer are stabilized by its positively charged C-terminus, which features two arginines that anchor to the bilayer and coordinate lipid phosphate groups. When IL binds to the bilayer, it largely resides in the interfacial region and its adsorption to the bilayer results in peptide desolvation. IL depletes the lipid density in its binding footprint, disrupting fatty acid tails of nearby lipids. These results are highlighted by a bilayer-aware clustering analysis, which shows that IL adopts dominant inserted and partially surface-bound states. We demonstrate that our simulation results are in good agreement with the available experimental data. Consequently, our simulations provide a complementary view of binding of IL to lipid bilayers that further elucidates its molecular mechanism.

## Graphical abstract

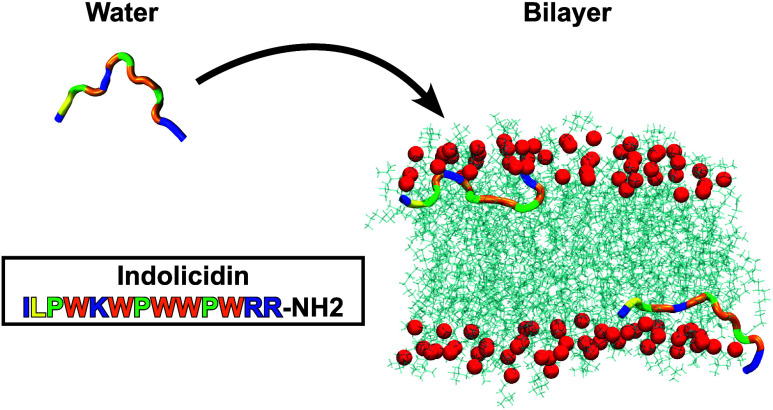

## Introduction

Antimicrobial peptides (AMPs) are produced by the innate immune system to combat infection.^1^ Although several mechanisms of AMP action have been proposed, their activity has largely been traced to their interactions with and disruption of anionic cell membranes.^1,2^ To date, thousands of AMP sequences have been identified across all biological kingdoms with sequences that are mostly cationic and approximately between 6 and 100 amino acids in length.^3^ Interest in AMPs is partially driven by rising antibiotic resistance, as AMP sequences can be optimized and used as alternatives to traditional antibiotics.^4^ Exploration of AMP sequences and their mechanisms of action has increasingly been pursued to develop the next generation of antibiotics and mitigate the growing threat of resistance.^5,6^

An AMP of focus is indolicidin (IL), which is found in cytoplasmic granules of bovine neutrophils.^7^ IL is part of the cathelicidin family and is known for its unique sequence (ILPWKWPWWPWRR-NH2), which contains 5 tryptophans, 3 prolines, and a +4 charge including its protonated N-terminus. IL has shown broad-spectrum activity against Gram-positive and Gram-negative bacteria, multidrug-resistant strains, biofilms, cancers, viruses, and fungi.^8^ IL toxicity has been attributed to its interactions with microbial cellular membranes, as well as with intracellular components such as DNA.^8–12^ IL has also induced cytotoxic effects in mammalian cells, resulting in hemolysis.^13^ Experiments have indicated that IL is active against both anionic and zwitterionic bilayers,^14,15^ although the precise physicochemical mechanism of IL activity remains unknown.^8^

CD spectra of IL in aqueous buffer show a disordered structure^13–15^ with some β-turn content.^15^ Similarly, CD spectra of IL in TFE show a disordered peptide structure.^13,15^ In the presence of sodium dodecyl sulfate (SDS) micelles, CD data have indicated that IL forms a turn, putatively due to indole ring stacking.^16^ NMR spectroscopy of IL in SDS or DPC has shown an extended structure with polyproline-II helix, with the latter forming a bend in the C-terminal producing a “boat-like” structure.^17^ When analyzed with the secondary structure assignment program STRIDE,^18^ NMR structures of IL in DPC (from PDB ID 1G89 ^17^) are annotated as random coil. These results together emphasize the overall unstructured chain of IL. Moreover, the placement of prolines along the sequence apparently drives this disordered structure, and replacing them with alanine results in the adoption of a helix.^19^

At low concentrations (≲3 μM), IL binding to a DPPC bilayer had no observable effect on the bilayer structure; however, at higher concentrations (>7.9 μM), IL eventually resulted in lysis.^20^ IL has been shown to bind to zwitterionic POPC large unilamellar vesicles (LUVs) with a free energy of transfer of −8.8 kcal/mol but binds approximately 3 kcal/mol more strongly to anionic LUVs containing POPG.^15^ In both cases, IL was able to induce leakage from LUVs, albeit more effectively in those containing POPG. In good agreement, IL exhibited approximately 2 kcal/mol greater binding affinity to LUVs composed of *Escherichia coli* polar lipid extract than to POPC LUVs.^21^ It has also been shown that IL binds more strongly to DMPC/DMPG vesicles with higher DMPG content, resulting in increased bilayer perturbation.^22^ The mechanism by which IL binds to lipid bilayers is thought to be driven by interactions between its tryptophans and lipids, placing IL at the bilayer interface.^17^ Although it has been suggested that IL activity is linked to its aggregation,^14,23^ IL has demonstrated an ability to induce leakage of POPG-containing LUVs even as a monomer.^15^

Molecular dynamics (MD) simulations of IL binding to DPC micelles show that it retains its NMR “boat-like” structure and forms π-cation interactions between Trp11 and Arg13.^24^ MD simulations have also been used to explore the adsorption of IL to lipid bilayers.^25–28^ Tsai et al. showed that IL is attracted to the POPC bilayer through electrostatic interactions between positively charged amino acids and negatively charged lipid phosphate groups.^26^ Hsu and Yip showed that IL retains its “boat-like” structure upon binding to several zwitterionic and anionic bilayer systems and that IL ultimately resides at the bilayer interface, resulting in bilayer thinning.^25^ Umbrella sampling MD simulations were also performed to study the adsorption of IL to DMPC or DMPC/DMPG bilayers on a quartz crystal surface, and these simulations demonstrated that IL has a higher binding affinity to anionic than to zwitterionic bilayers in good agreement with experiments.^27^ However, subsequent umbrella sampling simulations indicated that absolute free energy estimates of IL binding to bilayers are challenging to compute due to slow degrees of freedom associated with lipid reorganization around the adsorbing peptide.^28^

In the current work, replica-exchange molecular dynamics simulations with solute tempering (REST) are used to study the equilibrium ensemble of IL bound to the DMPC bilayer or in water. We have similarly applied REST simulations to uncover the binding profiles of Aβ and PGLa peptides to lipid bilayers.^29–31^ REST simulations of IL permit us to robustly investigate the structural changes that occur as IL binds and its interactions with the lipid bilayer. Our simulations have revealed that IL, which is largely unstructured, loses turn propensity upon binding to the bilayer compared to its structure in water. When IL binds to the bilayer, it forms stable contacts between its positively charged C-terminal arginines and lipid phosphate groups. Because of this strong anchoring, amino acids closer to the C-terminus are more likely to be inserted or surface-bound to the bilayer, whereas amino acids toward the N-terminus have a higher propensity of being unbound. IL is localized to the interfacial region between lipid headgroups and fatty acid tails, resulting in a local lipid density depression that reduces the thickness of the bilayer and perturbs lipids. We demonstrate that our results are consistent with available experimental data and, therefore, provide an atomistic view into the equilibrium ensemble of IL binding to the DMPC bilayer.

## Methods

### Simulation Setup

Replica-exchange molecular dynamics simulations with solute tempering (REST) were used to simulate the binding of indolicidin^7^ (IL) peptides with sequence ILPWKWPWWPWRR-NH2 to a dimyristoylphosphatidylcholine (DMPC) bilayer in explicit solvent (Figure 1). The DMPC bilayer was chosen because it is well-studied experimentally,^32^ providing a foundation for understanding the molecular mechanism of IL binding to lipid bilayers, and because it is relevant to IL interactions with mammalian cells, whose extracellular leaflets are rich in zwitterionic lipids.^33^ One IL was placed at each bilayer leaflet to capitalize on the inherent symmetry of the bilayer system and effectively double the sampling. This arrangement also ensures that numerical artifacts do not emerge along the pressure profile due to the periodic boundary conditions employed in simulations.^34^ With this design, the subsystem of interest consists of one IL peptide binding to a bilayer leaflet. Initial IL structures were taken from PDB ID 1G89 with a protonated N-terminus and amidated C-terminus.^17^ Peptides were placed around a 7 × 7 DMPC bilayer (49 lipids per leaflet; 98 lipids overall) in a simulation box initially of size 56.6 Å × 56.6 Å × 160 Å. Peptide and lipid atoms were represented using the CHARMM36m protein force field^35^ and CHARMM36 lipid force field,^36^ respectively. The system was solvated in 11,478 CHARMM-modified TIP3 water molecules.^37,38^ Due to the +4 positive charge on each IL peptide, 8 chloride counterions were added to neutralize the system charge. Additionally, REST simulations were performed of a single IL peptide solvated in 5585 CHARMM-modified TIP3 water molecules with 4 chloride counterions to neutralize the peptide charge.

**Figure 1:**
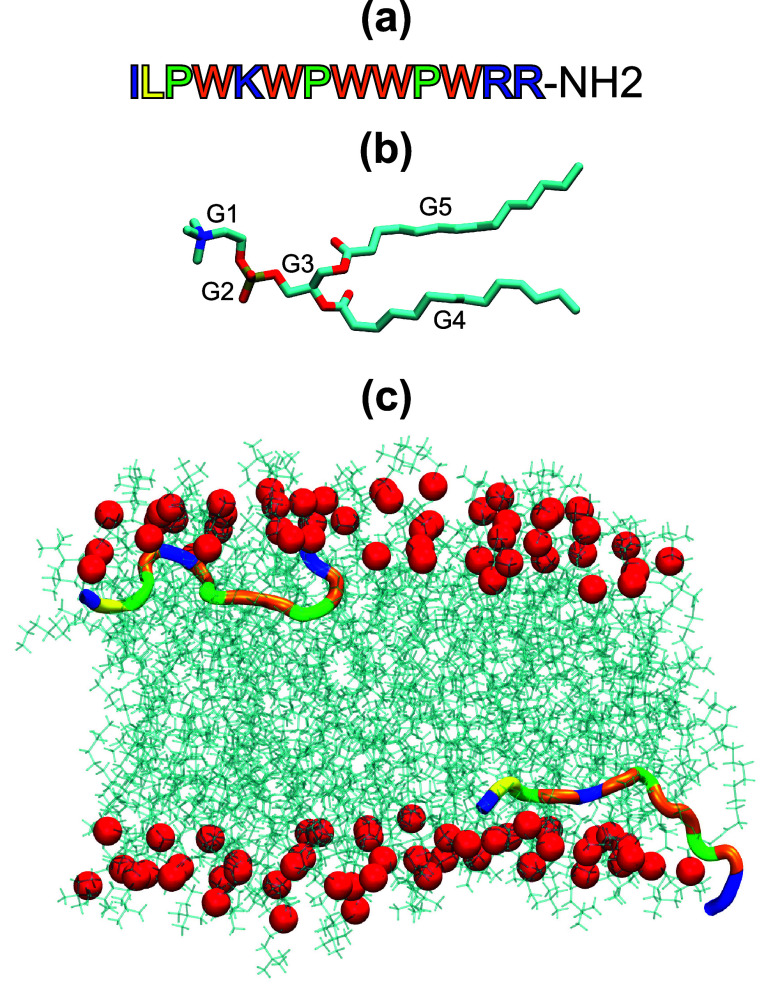
(a) IL sequence with positively charged amino acids (Ile1, Lys5, Arg12, and Arg13) in blue, Leu2 in yellow, Pro in green, and Trp in orange. (b) DMPC lipid with structural groups indicated where G1 corresponds to choline, G2 corresponds to the phosphate group, G3 is glycerol, and G4-G5 are fatty acid tails. (c) Depiction of our simulation setup with one IL peptide distributed on each bilayer leaflet. IL is colored according to panel (a). Lipids are represented by cyan lines, with their phosphorus atoms shown as red spheres.

Simulations were executed using the molecular dynamics (MD) program NAMD^39^ with periodic boundary conditions and an integration time step of 1 fs. Electrostatics were computed using particle-mesh Ewald summation, and van der Waals interactions were smoothly switched off over the interval from 8 to 12 Å, with the force switch option enabled to facilitate simulations with the bilayer. Temperature was controlled with Langevin dynamics setting the damping coefficient to 5 ps^–1^, and pressure was controlled with the Langevin piston method. For simulations involving the bilayer, pressure control followed a semi-isotropic scheme, where pressure in the (*x*, *y*) (bilayer plane) and *z* (bilayer normal) dimensions fluctuates independently. To preserve bilayer integrity at higher temperatures, harmonic restraints were applied to the *z* center of mass of lipid phosphorus (P) atoms in each leaflet with a target *z*_P_ = 17.4 Å and force constant *k* = 6.5 kcal/mol/Å^2^, matching our previous studies with DMPC.^29^ Importantly, the impact of these restraints is negligible with an average energy < *RT*. To maintain symmetry in our bilayer simulations, we also applied boundary restraints with *k* = 10 kcal/mol/Å^2^ to peptide atoms and counterions that came within 4 Å of the *z* periodic boundary, preventing them from crossing. These boundary restraints were rarely used with an average energy of ≪ *RT*.

REST was performed using *N*_R_ = 10 replicas for simulations of IL binding to the bilayer and *N*_R_ = 6 replicas for simulations of IL in water. In both cases, temperatures were geometrically distributed in the range from 330 to 430 K, and the pressure was held constant at 1 atm for all replicas. We selected the minimum temperature of 330 K to enhance conformational sampling and mitigate slow degrees of freedom associated with peptide insertion into the bilayer,^28^ which lead to poor convergence at lower temperatures.^28,31^ Several variants of REST exist,^40–44^ and we employed the variant that modifies replica enthalpies by applying the scaling factor $\sqrt{T_{r}/T_{0}}$ to solute–solvent energies and *T*_*r*_/*T*_0_ to solvent–solvent energies.^42,43^ To practically implement this REST variant for a given replica *r*, the thermostat used in simulations is set to replica temperature *T*_*r*_, but energy terms involving solvent are effectively quenched to the lowest temperature *T*_0_ = 330 K through REST scaling. REST is designed such that *T*_0_ is the temperature of interest, as REST scaling factors cancel out and the unmodified enthalpy is recovered only when *r* = 0. We defined the solute as IL peptides and counterions, and the solvent as lipids (if applicable for the given system) and water molecules. Replica exchange then follows the Metropolis criterion and exchanges between neighboring replicas *r* and *r* + 1 occur with the probability α = min­[1, e^–Δ^], where Δ = β_*r*_(*H*_*r*_(*X*_*r*+1_) – *H*_*r*_(*X*_*r*_)) + β_*r*+1_(*H*_*r*+1_(*X*_*r*_) – *H*_*r*+1_(*X*_*r*+1_)), β_*r*_ = (*R̅T*_*r*_)^−1^ with *R̅* as the gas constant, *H*_*r*_ is the enthalpy, and *X*_*r*_ is a system configuration for replica *r*. As explored previously by our group, the elimination of energy terms related to solvent from Δ due to REST scaling reduces the number of required replicas by 4- to 5-fold without significantly comprising sampling.^43^ Replica exchanges were attempted every 2 ps, and on average, this produced an exchange rate of 25.5% for the bilayer system and 29.1% for the system in water. For the bilayer system, we produced 6 trajectories, each starting from an independent MD run where IL was adsorbed to the bilayer. Each REST trajectory ran for a total of 300 ns per replica, or 3 μs over all replicas for a given trajectory. Considering the symmetric design of our system, the amount of cumulative sampling over all trajectories, replicas, and peptides is 36 μs. We produced 4 trajectories for simulations of IL in water, each of which had 200 ns per replica, or 4.8 μs over all trajectories and replicas. In both simulation systems, we reserved the last 50 ns of simulations from each trajectory for analysis. Evaluation of REST technical performance and simulation convergence is presented in the Supporting Information.

### Computation of Structural Probes

Peptide secondary structure was assigned using STRIDE.^18^ Intramolecular contacts *C* between peptide amino acids and intermolecular contacts *C*_*l*_ between amino acids and DMPC lipids or their structural groups *k* (Figure 1b) were determined if the distance between a pair of heavy atoms from two groups was less than 4.5 Å. Hydrogen bonds were assigned if the distance between donor (D) and acceptor (A) atoms was <3.5 Å and the angle ∠DHA > 120°. Peptide radius of gyration *R*_g_ was computed as the average distance of peptide heavy atoms from its geometric center, and the end-to-end distance *r*_1*N*_ was computed as the distance between the Cα atoms of Ile1 and Arg13.

To probe the binding profile of IL to the DMPC bilayer, we computed the probability *P*(*z*; *i*) for the side chain center of mass of amino acid *i* to be at the distance *z* from the bilayer midplane. We also computed the position *z*(*i*) of each amino acid *i* within the bilayer, as well as the probability for amino acid *i* to be inserted (*z*(*i*) < *z*_P_, where *z*_P_ is the center of mass of phosphorus (P) atoms within a leaflet), surface-bound (*z*_P_ ≤ *z*(*i*) < *z*_P_ + 6.5 Å), or unbound (*z*(*i*) ≥ *z*_P_ + 6.5 Å). The impact of IL on the bilayer structure was assessed by computing the lipid number density *n*_*l*_(*r*, *z*) as a function of the lipid heavy atom lateral distance from the peptide center of mass *r* and distance from the bilayer midplane *z*. In this profile, we defined a cutoff distance *r*_c_ = 13 Å at which IL contacts with lipids were found to be at a maximum. The thickness of the bilayer *D*(*r*) was defined by finding the distance *z* at which lipid and water densities are equal and then tracing this density along the *n*_*l*_(*r*, *z*) profile for the “upper” and “lower” leaflets. We then quantified the bilayer indentation caused by IL as the difference in thickness Δ*D* in regions near (*r* < *r*_c_) and away (*r* ≥ *r*_c_) from the IL binding footprint. Bilayer disordering due to IL binding was also measured by computing the lipid carbon-deuterium order parameter *S*_CD_ for *sn*-2 chains.^45^

Clustering of bilayer-bound IL was performed using the algorithm from Daura et al.^46^ and a bilayer-aware protocol for aligning structures and computing their root-mean-square deviation (RMSD). The alignment protocol is further described in the Supporting Information. Briefly, the *x* and *y* coordinates from two peptide structures extracted from our simulation trajectories are aligned using a two-dimensional (2D) implementation of the Kabsch algorithm.^47^ Peptide *z* positions are not subjected to translation or rotation during alignment to preserve the peptide insertion depth. After alignment, RMSD is computed between the two peptides using their *x*, *y*, and *z* coordinates to report the difference in peptide poses with respect to their insertion into the bilayer given that insertion depth is unaltered by this protocol. Pairwise alignment and RMSD calculations were performed using every 10th structure from our simulation trajectories.

VMD^48^ and MDAnalysis^49^ supplemented with custom Python and Fortran scripts were used to conduct analyses. We denote thermodynamic quantities by angular brackets ⟨···⟩. All quantities, unless otherwise noted, are computed at the temperature of interest *T*_0_ = 330 K. Standard errors are computed considering each simulation trajectory as an independent sample.

## Results and Discussion

### Indolicidin Structure

We performed all-atom replica-exchange molecular dynamics simulations with solute tempering (REST) to investigate the structure of indolicidin (IL) when bound to the DMPC bilayer or in water. Our REST simulations involving the DMPC bilayer strictly sample the bound state of IL with a binding probability of 0.98 ± 0.01. To characterize the IL structure, we computed the secondary structure profile according to STRIDE.^18^ Overall, DMPC-bound IL residues are composed of random coil (⟨RC⟩ = 0.80 ± 0.02) followed by turn (⟨*T*⟩ = 0.20 ± 0.01). Propensities for other secondary structure types are negligible (<0.01). Because the IL secondary structure is dominated by random coil and turn, in Figure 2, we present the turn propensity ⟨*T*(*i*)⟩ for each amino acid *i* in the IL sequence. Moderate turn (⟨*T*(*i*)⟩ ≥ 0.25) occurs for interior amino acids Pro3-Trp6 and Trp9. We compare these results to those from our simulation of IL in water, where the random coil propensity is slightly suppressed (⟨RC⟩ = 0.68 ± 0.05) while the turn propensity is elevated (⟨*T*⟩ = 0.31 ± 0.04). In the water environment, ⟨*T*(*i*)⟩ ≥ 0.25 for Pro3-Trp9, all with turn propensities that surpass their respective propensities in the bilayer and with Trp4-Trp6 exceeding 0.5. IL turn propensity can be further explored by computing the average number of β-and γ-turns, *n*_β_ and *n*_γ_, respectively, as reported by STRIDE. In both the bilayer and water environments, *n*_β_ exceeds *n*_γ_, with corresponding values of *n*_β_ = 0.60 ± 0.06 vs *n*_γ_ = 0.12 ± 0.01 for the bilayer environment and 1.16 ± 0.22 vs 0.08 ± 0.01 for the water environment, i.e., in water, there are almost 2-fold more β-turns than in the bilayer environment. Taken together, these results demonstrate that IL is largely unstructured in both bilayer and water environments and that binding to the bilayer induces IL disorder, decreasing turn content overall by 36%. These findings are qualitatively consistent with experimental data, which have shown a largely disordered structure with some β-turn content.^13–17^ Interestingly, loss of secondary structure upon IL binding to the bilayer contrasts with AMPs that gain helix during binding.^4^ Based on the Wimley-White hydrophobicity scale,^50^ IL has a hydrophobic moment of 3.79 Å·kcal/mol, which is similar to the hydrophobic moments of helical peptides.^29,51^ For IL, the lack of helix is putatively due to the presence of Pro, and when Pro are substituted with Ala, the peptide adopts a helical structure^19^ with a hydrophobic moment of 3.58 Å·kcal/mol. Pro amino acids are then expected to be responsible for the secondary structure profile observed in our simulations. Nisin, another AMP, appears to exhibit similar behavior, where lanthionine rings suppress backbone fluctuations and contribute to the peptide’s unstructured state, particularly in its pore-forming C-terminal residues 23–34.^52^ By replacing nisin C-terminal residues Abu and Dha with Ala, we computed a hydrophobic moment of 4.56 Å·kcal/mol, hinting that this peptide may also have a hidden helix propensity that is masked by its backbone-stabilizing lanthionine bridges.

**Figure 2:**
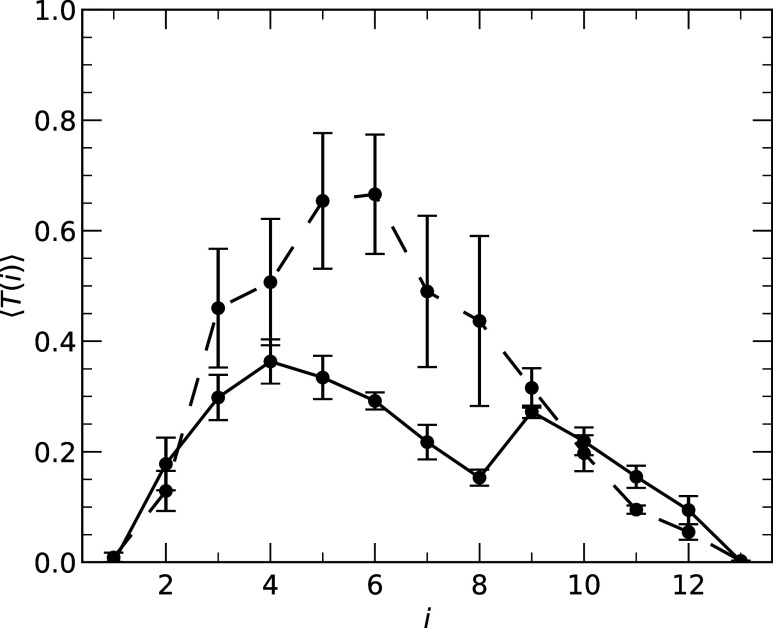
Probability of turn ⟨*T*(*i*)⟩ for each amino acid *i* in the IL sequence computed for IL bound to the DMPC bilayer (solid line) and in water (dashed line). Vertical bars show the standard errors.

IL tertiary structure was assessed by computing the probability of forming intrapeptide contacts ⟨*C*(*i*, *j*)⟩ between residues *i* and *j* where |*j* – *i*| ≥ 2. In agreement with the secondary structure profile, the top contacts where ⟨*C*(*i*, *j*)⟩ > 0.5 occur only for turn-forming contacts |*j* – *i*| = 2 (Table 1). When IL is bound to the DMPC bilayer or in water, the top three contacts are formed with a probability of 1.0 and are identical in composition, albeit with a different order due to small numerical differences (<0.001). The order of the rest of the top contacts, excluding the top three, shares minor similarity between bilayer and water environments with a Spearman’s rank correlation coefficient of 0.26. (For comparison, including the top three yields a coefficient of 0.69). Although partially expected due to the high representation of Trp in the IL sequence (∼39% of residues) and the bulkiness of Trp (∼50% of all IL heavy atoms), top intrapeptide contacts involving Trp tend to have a higher contact probability (0.92 in the bilayer vs 0.94 in water) than those without Trp (0.81 vs 0.88). Furthermore, the top 5 contacts all involve Trp, and we can conclude that Trp is dominant in defining the IL tertiary structure. For comparison, the top intrapeptide contacts computed for the PDB structure 1G89, which contains IL in the presence of DPC micelles, show all |*j* – *i*| = 2 contacts except Pro10-Arg12 formed in its 16 NMR conformers and is thus in qualitative agreement with our findings.^17^

**Table 1:** Top Intrapeptide Contacts

rank	⟨*C*_bilayer_(*i*, *j*)⟩^a^	⟨*C*_water_(*i*, *j*)⟩^a^
1	Trp6-Trp8 (1.00 ± 0.00)	Trp9-Trp11 (1.00 ± 0.00)
2	Trp9-Trp11 (1.00 ± 0.00)	Leu2-Trp4 (1.00 ± 0.00)
3	Leu2-Trp4 (1.00 ± 0.00)	Trp6-Trp8 (1.00 ± 0.00)
4	Trp11-Arg13 (0.95 ± 0.02)	Trp4-Trp6 (0.95 ± 0.02)
5	Trp8-Pro10 (0.93 ± 0.02)	Trp11-Arg13 (0.92 ± 0.03)
6	Ile1-Pro3 (0.89 ± 0.01)	Ile1-Pro3 (0.91 ± 0.01)
7	Trp4-Trp6 (0.87 ± 0.04)	Pro3-Lys5 (0.88 ± 0.02)
8	Lys5-Pro7 (0.83 ± 0.02)	Pro10-Arg12 (0.87 ± 0.02)
9	Pro10-Arg12 (0.82 ± 0.02)	Pro7-Trp9 (0.87 ± 0.01)
10	Pro7-Trp9 (0.72 ± 0.05)	Trp8-Pro10 (0.85 ± 0.06)
11	Pro3-Lys5 (0.70 ± 0.06)	Lys5-Pro7 (0.84 ± 0.03)

a ⟨*C*_bilayer_(*i*, *j*)⟩ and ⟨*C*_water_(*i*, *j*)⟩ correspond to ⟨*C*(*i*, *j*)⟩ for IL bound to the bilayer and in water, respectively.

The change in tertiary structure Δ*C*(*i*, *j*) = ⟨*C*_bilayer_(*i*, *j*)⟩ – ⟨*C*_water_(*i*, *j*)⟩ between the bilayer and water environments (Figure 3) shows that IL is more structured in water than when bound to the bilayer with Δ*C*(*i*, *j*) < 0 for ∼73% of contact pairs |*j* – *i*| ≥ 2. All 10 contact pairs with |Δ*C*(*i*, *j*)| ≥ 0.1 are disrupted in the bilayer, i.e., ⟨*C*_bilayer_(*i*, *j*)⟩ < ⟨*C*_water_(*i*, *j*)⟩ (Table S1). Of these contacts, only Pro3-Lys5 and Pro7-Trp9 are listed as top contacts in Table 1, highlighting the importance of these amino acids in defining the IL tertiary fold. Moreover, the average Δ*C* = −2.1 ± 0.8 Å, which illustrates the overall destabilization of the tertiary structure when IL is bound to the bilayer. Similar conclusions can be made when looking at other structural quantities, such as the radius of gyration *R*_g_ and end-to-end distance *r*_1*N*_. For the former, in the bilayer environment ⟨*R*_g_⟩ = 9.7 ± 0.2 Å but collapses to 9.0 ± 0.3 Å in water. The end-to-end distance ⟨*r*_1*N*_⟩ also shows peptide extension upon binding to the bilayer (23.9 ± 0.8) versus in water (21.5 ± 1.2 Å). IL therefore forms more intrapeptide contacts in water making the peptide more compact, whereas in the bilayer environment the peptide becomes expanded. These findings corroborate those presented in our secondary structure analysis, where the loss in the IL structure in the presence of the bilayer was attributed to a decrease in turn and increase in random coil content.

**Figure 3:**
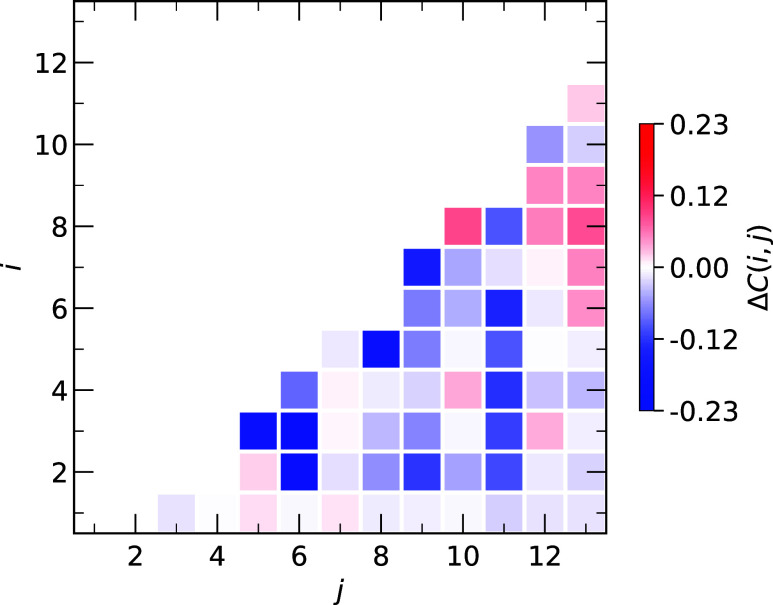
Change in tertiary contacts Δ*C*(*i*, *j*) between IL residues *i* and *j* when bound to the DMPC bilayer or in water. Strictly, Δ*C*(*i*, *j*) = ⟨*C*_bilayer_(*i*, *j*)⟩ – ⟨*C*_water_(*i*, *j*)⟩. The color scale shows the value of Δ*C*(*i*, *j*) and is symmetric around 0.

### Indolicidin Interactions with DMPC

Our simulations of IL binding to the DMPC bilayer can be probed to examine interactions between the peptide and lipids. In Figure 4, we plot the probability *P*(*z*; *i*) for the side chain center of mass of amino acid *i* to be at the distance *z* from the bilayer midplane. This profile shows that the most stable interactions occur between lipid headgroups and Arg12 and Arg13, which are anchored at approximately *z*_P_ via their electrostatic interactions with negatively charged lipid phosphate groups. Additionally, Figure 4 shows that IL adopts a well-defined inserted state below *z*_P_, although surface-bound and unbound states are also sparsely sampled. To further quantify these states, we computed the probability for an amino acid *i* to be inserted *P*_*i*_, surface-bound *P*_s_, and unbound *P*_u_ (see Methods Section). Table S2 shows that all amino acids *i* feature an insertion probability *P*_*i*_(*i*) > 0.5 except for *i* = Lys5, and on average *P*_*i*_ over all amino acids is 0.57. For comparison, the average probability of surface-bound *P*_s_ and unbound *P*_u_ is 0.20 and 0.23, respectively. *P*_s_(*i*) and *P*_u_(*i*) indicate that, when not inserted, N-terminal amino acids Ile1-Trp8 favor an unbound state with *P*_u_(*i*) > *P*_s_(*i*), while C-terminal amino acids Pro10-Arg13 favor the surface-bound state with *P*_s_(*i*) > *P*_u_(*i*) due to the strong stabilizing interactions of Arg12 and Arg13 with the bilayer.

**Figure 4:**
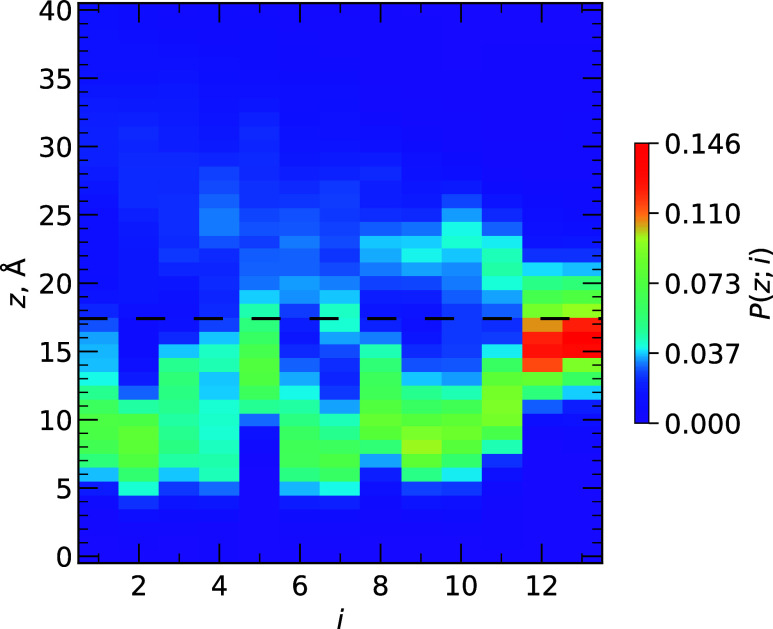
Probability distributions of the side chain centers of mass for amino acid *i* as a function of their distance *z* from the bilayer midplane. The color scale on the right shows the probability *P*(*z*; *i*) as a function of *z* given *i*. The dashed line shows *z*_P_.

Experiments with NMR and fluorescent spectroscopy have shown that IL is located in the bilayer’s interfacial region,^15,17,21^ in agreement with our simulations that sample IL with an average ⟨*z*⟩ = 17.8 ± 1.1 Å near *z*_P_ and lipid headgroups. Andrushchenko et al.^21^ indicated that IL is shielded from water when bound to phosphatidylcholine large unilamellar vesicles (LUVs) resulting in a 35% change in Stern–Volmer constants, which measure acrylamide quenching in the bilayer compared to buffer. A similar estimate of solvent accessibility can be directly computed from our simulation ensembles by considering the number of water molecules in contact with IL in water (171.5 ± 2.9) or bound to the bilayer (95.3 ± 6.2), i.e., a 44% change in solvent accessibility comparable to the experimental value. Finally, experiments of IL interacting with POPC LUVs have also determined that Trp residues are located ∼11 Å from the bilayer midplane.^15^ We computed in Figure S5 the distribution of *z* for all Trp residues, which have their most prominent peak at 10 Å. Collectively, our simulations are therefore in good agreement with experiments, indicating an overall consistent IL binding profile.

Peptide binding to lipid atoms can be explored in more detail by computing the average number ⟨*C*_*l*_(*i*)⟩ of lipids in contact with IL amino acids *i* (Figure 5a). C-terminal amino acids Trp11-Arg13 all coordinate 3 lipids, whereas all other Trp and N-terminal amino acids Ile1 and Leu2 coordinate more than 2 lipids on average. Lipid binding is subdued in the remaining amino acids, which include all Pro and Lys5. Interestingly, Arg12 and Arg13 coordinate 1.3 ± 0.1 lipids in tandem; i.e., approximately 40% of lipid contacts made by these amino acids are shared between them. High degree of lipid coordination in the C-terminus involves the formation of hydrogen bonds, which we assessed by computing the average number of contacts that include hydrogen bonds ⟨*C*_*l*,hb_(*i*)⟩ (Figure 5a). Indeed, 61 and 66% of the Arg12 and Arg13 contacts in a given structure involve at least 1 hydrogen bond. Elsewhere in the sequence, hydrogen bonds are modestly seen for positively charged Lys5 (46% ⟨*C*_*l*_(5)⟩) and Ile1 (37% of ⟨*C*_*l*_(1)⟩). To further probe the interactions between amino acids and lipids, we computed the average number ⟨*C*_*l*_(*i*, *k*)⟩ of lipid structural groups *k* (see Figure 1b) in contact with IL amino acids *i* (Figure 5b). We considered a lipid structural group *k* in contact with amino acid *i* as stable if ⟨*C*_*l*_(*i*, *k*)⟩ ≥ 1. For lipid headgroups, which include choline G1, phosphate groups G2, and glycerol G3, we observe stable contacts between Arg12-G1, Arg13-G1, Ile1-G2, Lys5-G2, Trp11-G2, Arg12-G2, Arg13-G2, Trp11-G3, Arg12-G3, and Arg13-G3. Out of these contacts, Arg12 and Arg13 interactions with G2 are the strongest, with these amino acids coordinating over 2 lipid G2 groups on average. Stable contacts with fatty acid tails G4 or G5 occur for Ile1, Trp4, Trp6, Trp8, Trp9, Trp11, and Arg13. Collectively, these results emphasize that the strongest interactions between IL amino acids and lipids occur through the C-terminal charged Arg12 and Arg13 binding to multiple lipids, largely through electrostatic contacts with phosphate groups G2 that are mediated by hydrogen bonding. Previous Ala-scanning experiments have also explored the importance of these two terminal Arg, and it was found that replacing either Arg12 or Arg13 with Ala increased the minimum inhibitory concentration of IL against *Staphylococcus aureus* or *E. coli* by more than 3-fold.^53^

**Figure 5:**
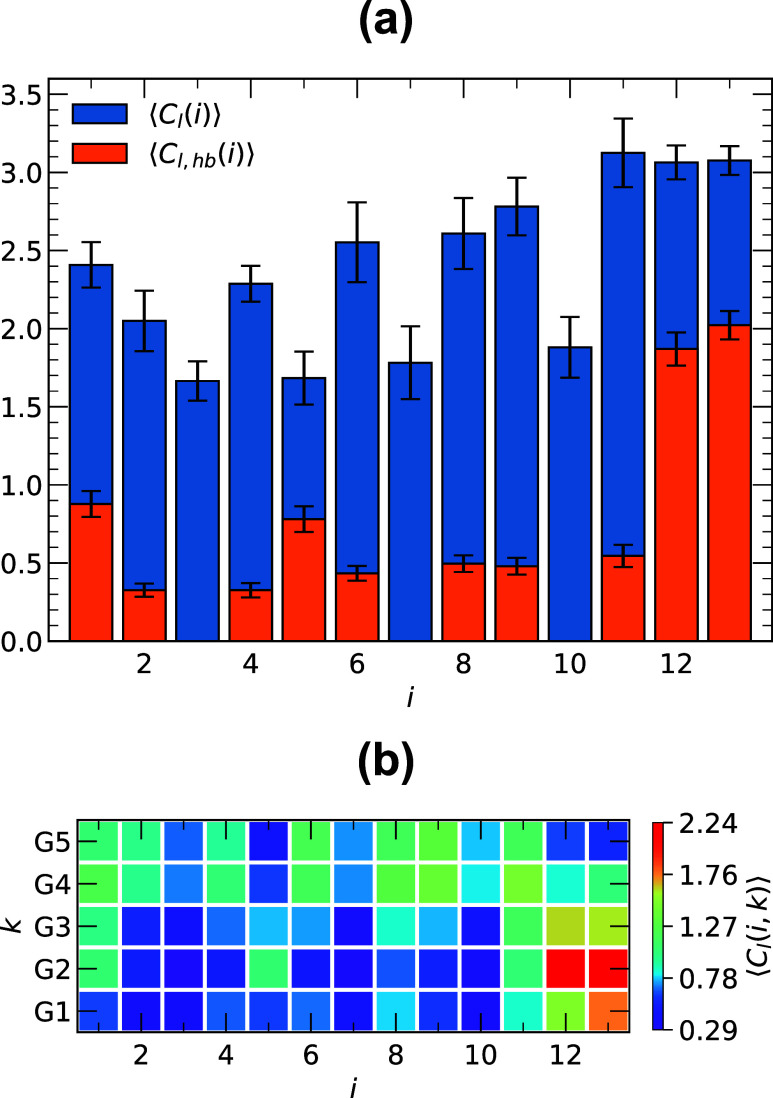
(a) Number of lipid contacts ⟨*C*_*l*_(*i*)⟩ with amino acids *i* are shown in blue, and the number of lipid contacts involving hydrogen bonding ⟨*C*_*l*,hb_(*i*)⟩ with amino acids *i* are shown in orange. Vertical bars indicate standard errors. (b) Number of lipid contacts ⟨*C*_*l*_(*i*, *k*)⟩ between lipid structural groups *k* and amino acids *i*. The color scale on the right shows the magnitude of ⟨*C*_*l*_(*i*, *k*)⟩.

### Indolicidin Effect on the Bilayer Structure

Since IL is predominately inserted below *z*_*P*_, we sought to quantify its effect on the lipid bilayer structure. We computed the average number density of lipid heavy atoms ⟨*n*_*l*_(*r*, *z*)⟩ as a function of the lateral distance *r* from the peptide center of mass and the distance *z* from the bilayer midplane (Figure 6). In the “upper” leaflet, which considers the bound peptide, there is a lipid density depression, i.e., with lipid atoms pushed away and space filled by peptide atoms. For regions that are away from the peptide when *r* ≥ *r*_c_ (see Methods Section), the average density is 0.033 ± 0.000 Å^–3^, which drops by 21% to 0.026 ± 0.001 Å^–3^ for near lipids when *r* < *r*_c_ indicating a thinning of the membrane. For comparison, the “lower” leaflet is virtually insensitive to the peptide in the opposing “upper” leaflet and the density is 0.033 ± 0.000 Å^–3^ in near and away regions of the bilayer. From ⟨*n*_*l*_(*r*, *z*)⟩, we also computed the thickness of the bilayer leaflet *D*(*r*) as a function of the lateral distance *r* from the peptide center of mass. In the away region, average *D*(*r* ≥ *r*_c_) = 37.9 ± 0.0 Å, which is reduced by 7% to 35.2 ± 0.5 Å in the near region. However, the density drop-off continues as *r* approaches 0 Å, and *D*(*r* ≈ 0) = 26.0 ± 1.5 Å. As a result, the largest change in thickness Δ*D* is 11.9 ± 1.5 Å or a 69% reduction.

**Figure 6:**
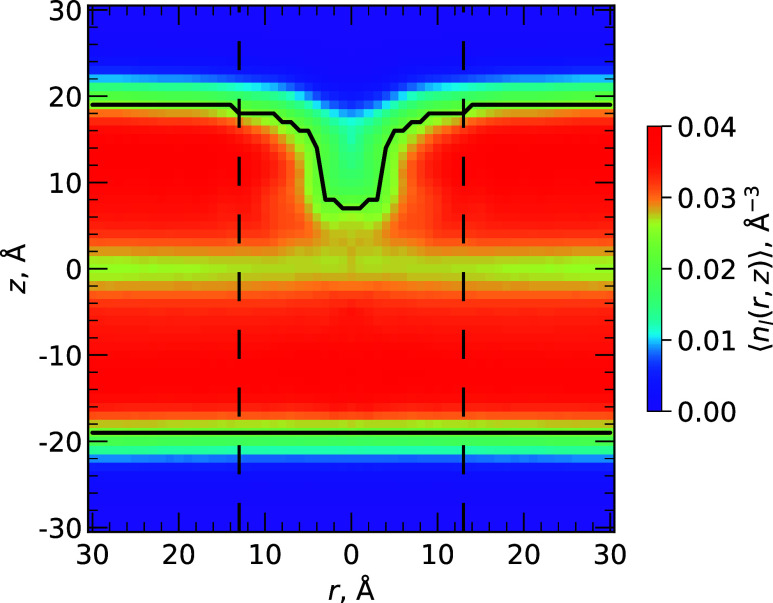
Density of lipid heavy atoms ⟨*n*_*l*_(*r*, *z*)⟩ as a function of the lateral distance *r* from the peptide center of mass and distance *z* from the bilayer midplane. Strictly, ⟨*n*_*l*_(*r*, *z*)⟩ was computed for each peptide in its given leaflet and averaged together, keeping the peptide in the “upper” leaflet while the “lower” leaflet marginalizes the opposing peptide. The black line represents profile *D*(*r*), which shows the indentation of the bilayer due to the peptide. Dashed vertical lines show the boundary *r*_c_ = 13 Å, representing the extent to which lipid atoms are interacting with IL. The color scale shows the magnitude of the density.

Creation of a local lipid density depression by IL is expected to affect the ordering of lipid fatty acid tails. We computed the lipid carbon-deuterium order parameter ⟨*S*_CD_(*i*)⟩ for each carbon *i* in *sn*-2 fatty acid tails (Figure 7), which shows that lipids in contact with IL are disordered compared to lipids not in contact. Average –⟨*S*_CD_⟩ over all carbons is 0.160 ± 0.002 for lipids not in contact with IL, and this is reduced by 13% to 0.140 ± 0.002 for lipids in contact. Taken together, the data in Figures 6 and 7 demonstrate that IL insertion into the lipid bilayer both indents the bilayer and disorders the lipid fatty acid tails.

**Figure 7:**
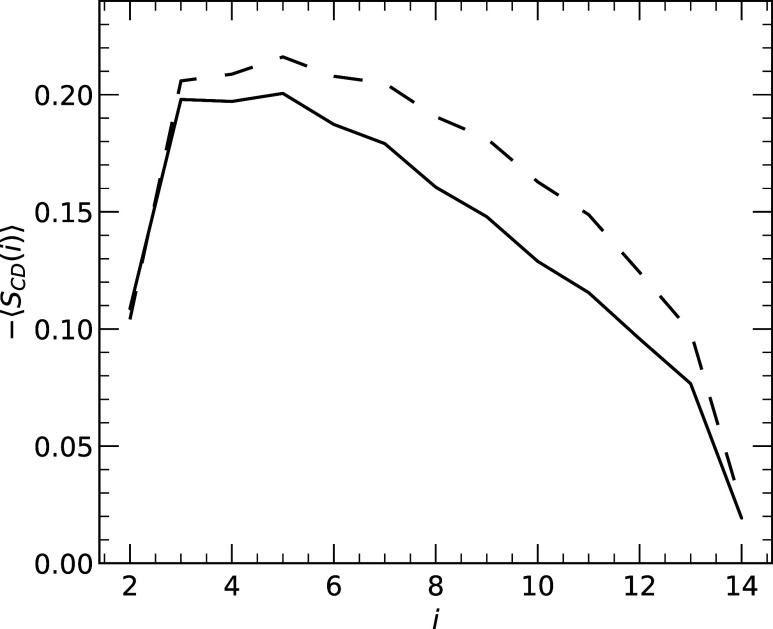
Lipid carbon-deuterium order parameter ⟨*S*_CD_(*i*)⟩ computed for each carbon *i* in the lipid *sn*-2 fatty acid chains. The solid line is computed for lipids in contact with IL, whereas the dashed line is for lipids not in contact. Standard errors are not shown because they are below 0.01.

### Indolicidin Binding Mechanism to DMPC

To summarize our simulation results, we performed bilayer-aware clustering using a 2D implementation of the Kabsch algorithm^47^ and the clustering algorithm from Daura et al.^46^ as described in the Methods Section. To ensure our clustering results assigned the vast majority of structures to clusters, we set the RMSD cutoff to 5.4 Å and captured >90% of structures in 17 populated clusters, whose probabilities of occurrence exceed 1%. The centroids for populated clusters in Figure 8 show that clusters sample inserted, surface-bound, and unbound amino acid states. The diversity of poses is also shown in Figure S6, which presents the average distance ⟨*z*(*i*; *c*)⟩ of each amino acid *i* center of mass from the bilayer midplane for each populated cluster *c*. In all clusters, the C-terminus is largely bound at approximately *z*_P_ supporting our conclusions from Figure 5 that Arg12 and Arg13 are important anchors to lipid phosphate groups.

**Figure 8:**
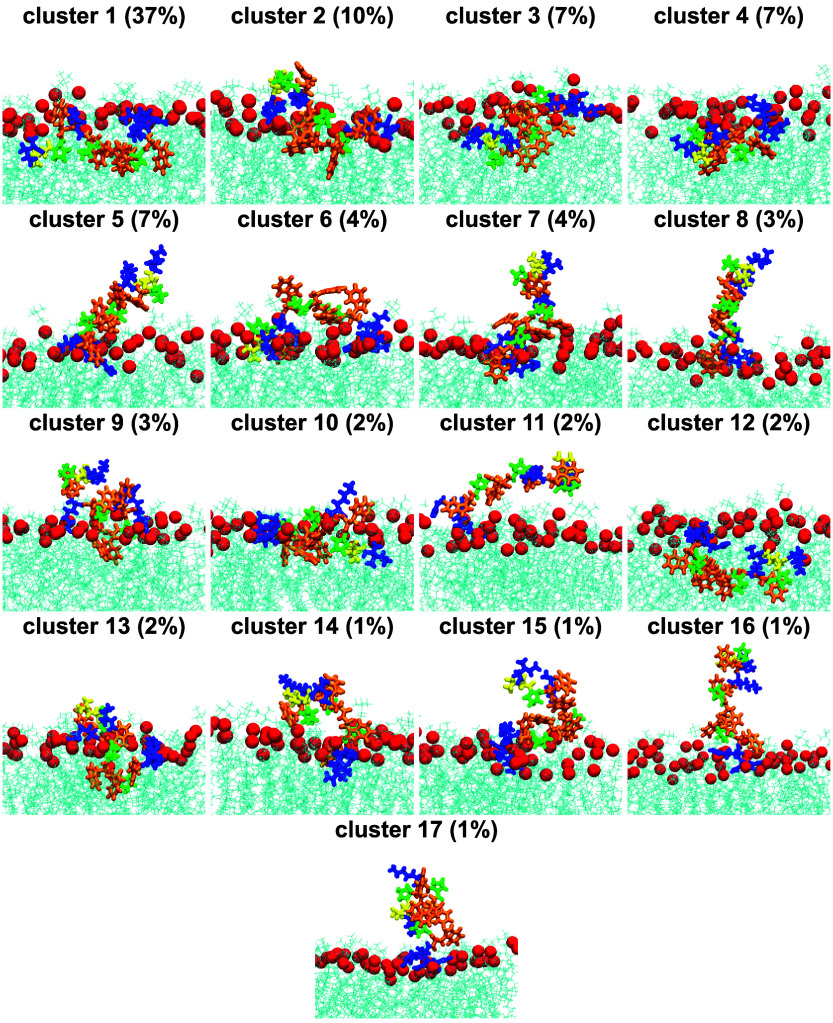
Centroids of populated clusters of IL bound to the DMPC bilayer. Cluster probabilities of occurrence are given in parentheses. Peptide amino acids are shown in licorice representation and are colored to match Figure 1a. Lipids are shown as cyan lines with phosphorus atoms as red spheres.

Out of all populated clusters, only clusters 1 and 2 have probabilities of occurrence >10%, and together these two clusters comprise almost 50% of structures. Analysis of Figure S6 reveals that cluster 1 is inserted and cluster 2 is partially surface-bound, thereby affording the opportunity to compute the difference in structural profiles between these two states. For cluster 1, the average turn content ⟨*T*(*c* = 1)⟩ is 0.14, which increases more than 2.4-fold to ⟨*T*(*c* = 2)⟩ = 0.34 in cluster 2. This increase in turn coincides with an overall compaction of the IL chain, increasing the number of intrapeptide contacts ⟨*C*(*c*)⟩ by 19% from 12.5 to 14.9, decreasing the radius of gyration ⟨*R*_g_(*c*)⟩ by 8% from 10.1 to 9.3, and decreasing the end-to-end distance ⟨*r*_1*N*_(*c*)⟩ by 18% from 25.6 to 21.1 Å. As a result, the partially surface-bound state in cluster 2 is similar to the structure of IL in water, with ⟨*T*⟩ = 0.31, ⟨*C*⟩ = 15.4 Å, ⟨*R*_g_⟩ = 9.0 Å, and ⟨*r*_1*N*_⟩ = 21.5 Å. We also computed the impact of clusters on the maximal change in bilayer thickness Δ*D* and found that both clusters 1 and 2 exceed the ensemble average of 11.9 Å, with Δ*D* of 14.8 and 12.9 Å, respectively, resulting in a density depression in the bilayer. The impact of this density depression is also felt by lipid fatty acid tails; the relative decrease in –⟨*S*_CD_(*c*)⟩ between lipids not in contact or in contact with IL is 17% for cluster 1 and 13% for cluster 2. As expected, the inserted state observed in cluster 1 thus has a more profound impact on bilayer structural properties than the partially surface-bound state in cluster 2.

In summary, our clustering analysis reveals distinct poses that contribute to the molecular mechanism of binding of IL to the DMPC bilayer. All populated clusters show the hallmark electrostatic interaction that occurs between positively charged C-terminal amino acids Arg12 and Arg13 with lipid headgroups at approximately *z*_P_. This anchoring of the C-terminus is in contrast to the N-terminus, which samples a diversity of bound states across clusters without a dominant pose. Upon insertion, our clustering analysis shows IL losing its structure from the water environment and adopting a more disordered and elongated conformation while indenting and disrupting the lipid bilayer. Our simulations therefore provide a mechanistic view into IL binding modes.

## Conclusions

We implemented REST simulations to probe the binding of IL to the DMPC bilayer, assess the differences in its structural ensemble with a water environment, investigate peptide-lipid interactions, and describe the impact of IL on the bilayer structure. Our simulations demonstrate that IL, which is predominately in a random coil state with some turn, has more turn structure in water than when bound to the bilayer. The loss in structure upon binding to the bilayer is also evident from a reduction in tertiary contacts and a corresponding conformational expansion, as indicated by increases in radius of gyration *R*_g_ and end-to-end distance *r*_1*N*_. IL binds to the lipid bilayer in its interfacial region, at approximately the same *z* position as lipid phosphorus atoms. The most stable interactions between IL and the bilayer come from its C-terminal amino acids Arg12 and Arg13, which coordinate more than 3 lipids on average and provide an anchor for binding. Due to this anchoring, other amino acids near the C-terminus are more likely to be inserted or surface-bound, whereas amino acids near the N-terminus have a higher likelihood of being unbound from the bilayer. IL binding results in a minor indentation of up to 11.9 Å of the bilayer surface, consistent with previous evidence of membrane thinning, and this indentation is sufficient to result in a local decrease in lipid fatty acid tail ordering. Moreover, our bilayer-aware clustering analysis revealed the dominant inserted and partially surface-bound states of IL, showing that on the surface, IL exhibits conformations similar to those seen in a water that become lost upon insertion. Our REST simulations provide good agreement with previous experimental data. Overall, our work provides an in-depth investigation into the equilibrium structural ensemble of IL when bound to DMPC bilayers, which is important to provide mechanistic explanations for the behavior of this unusual unstructured AMP.

## Supplementary Material



## Data Availability

NAMD is available at https://www.ks.uiuc.edu/Research/namd/. VMD is available at https://www.ks.uiuc.edu/Research/vmd/. Initial structures, topology files, and NAMD configuration files are available at https://github.com/LockhartLab/IL_DMPC. Codes used for data analysis are available from the authors upon request.

## Notes

It provides details concerning the model and methods used as well as additional data (PDF)
