# Supplementary material for: Binding of Antimicrobial Peptide Indolicidin to DMPC Bilayer Using Replica-Exchange Molecular Dynamics
Source: J Chem Inf Model. 2025 Aug 21;65(17):9251–60. doi: 10.1021/acs.jcim.5c01153 (PMC12421676; doi:10.1021/acs.jcim.5c01153)
Supplement: Supplementary file 1 [file ci5c01153_si_001.pdf]

Supporting Information

Binding of antimicrobial peptide indolicidin to DMPC bilayer using replica-exchange molecular dynamics

Alex R. Fitz, Dmitri K. Klimov, and Christopher Lockhart*

School of Systems Biology, George Mason University, Manassas, VA 20110

E-mail: clockha2@gmu.edu

REST Technical Performance

The technical performance of replica-exchange molecular dynamics simulations with solute tempering (REST) was assessed by computing exchange rates and analyzing replica mixing. We chose the number of replicas ($N_R = 10$ for indolicidin (IL) binding to the DMPC bilayer and $N_R = 6$ for IL in bilayer-free water) across the temperature scale of 330 to 430 K to produce an exchange rate between 20-30% following our previous studies.[1] We computed an average exchange rate α of 25.5% ± 0.1% for simulations of IL bound to the bilayer and 29.1% ± 0.1% for IL in water. Figs. S1a and S1b show that exchange rates $\alpha(T)$ are close to these average values across all temperatures T and are within the desired range.

Replica mixing was quantified using the mixing parameter from Han and Hansmann.[2] For a given temperature T, the mixing parameter $m(T) = 1 - \frac{\sqrt{\sum_{r=1}^{N_R} t_r^2}}{\sum_{r=1}^{N_R} t_r}$, where t_r is the amount of simulation time that a replica r spends at temperature T. If N_R replicas are equally represented at a given T, the mixing parameter approaches a theoretical maximum of $m^* = 1 - 1/\sqrt{N_R}$. For simulations of IL binding to the DMPC bilayer, we found that the

average $m(T)$ is 0.65 ± 0.01 and therefore close to $m^* = 0.68$ for $N_R = 10$ replicas (Fig. S1c). Simulations of IL in water have an average $m(T) = 0.59 \pm 0.00$, which perfectly agrees with m^* for $N_R = 6$ (Fig. S1d). Comparing the two $m(T)$ profiles, bowing of $m(T)$ at the upper and lower ends of the temperature schedule for bilayer simulations is related to slower mixing of replicas across temperatures. However, both profiles indicate that $m(T)$ approaches m^*, indicating that sufficient mixing has occurred.

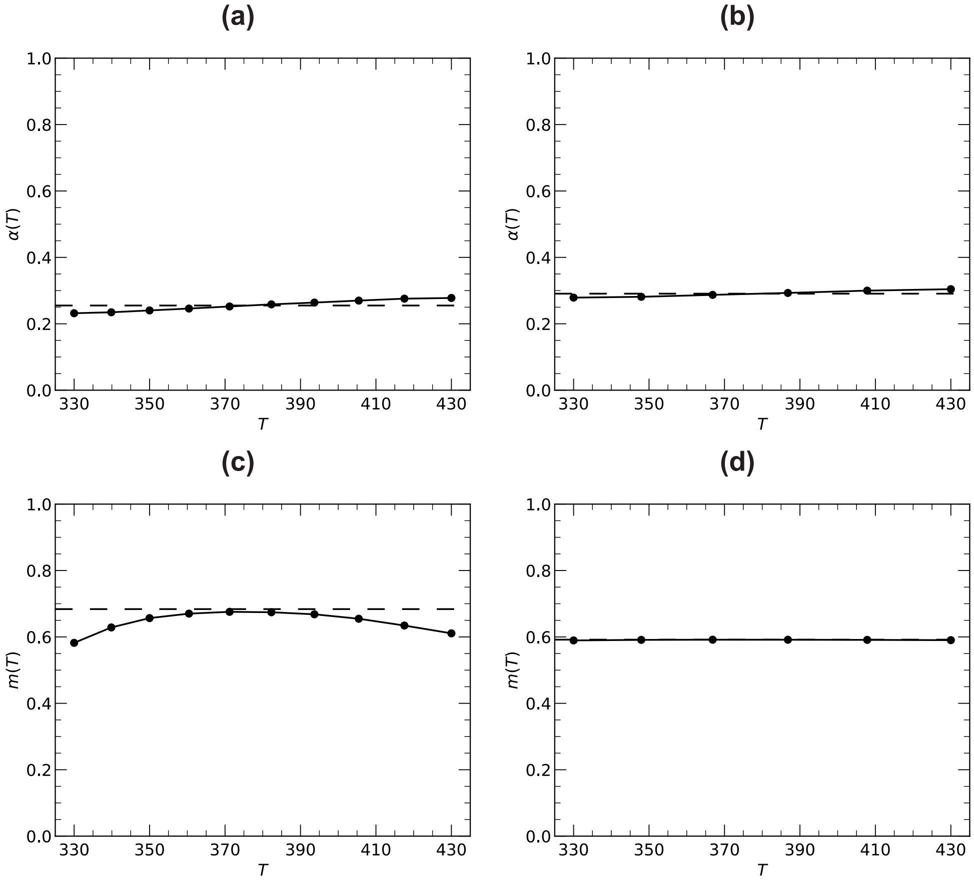

Fig. S1. Exchange rates $\alpha(T)$ for each temperature T are shown for simulations of IL (a) binding to the DMPC bilayer and (b) in water. In both systems, exchange rates across all temperatures are close to their average values, denoted by horizontal dashed lines. Replica mixing parameters $m(T)$ for each temperature T are shown for IL (c) binding to DMPC and (d) in water. Both systems approach theoretical m^*, denoted by horizontal dashed lines.

To illustrate successful mixing within a simulation trajectory, we plotted the random walk of replicas across temperatures in Fig. S2. If replicas are well exchanged, this is expected to produce a color mosaic, indicating that replicas are not significantly trapped at any given

condition and instead walk unencumbered across the temperature schedule. Indeed, this is observed for both simulation systems of IL binding to the DMPC bilayer and in water. Taken together, these metrics indicate that REST is technically performing as expected for our simulation systems.

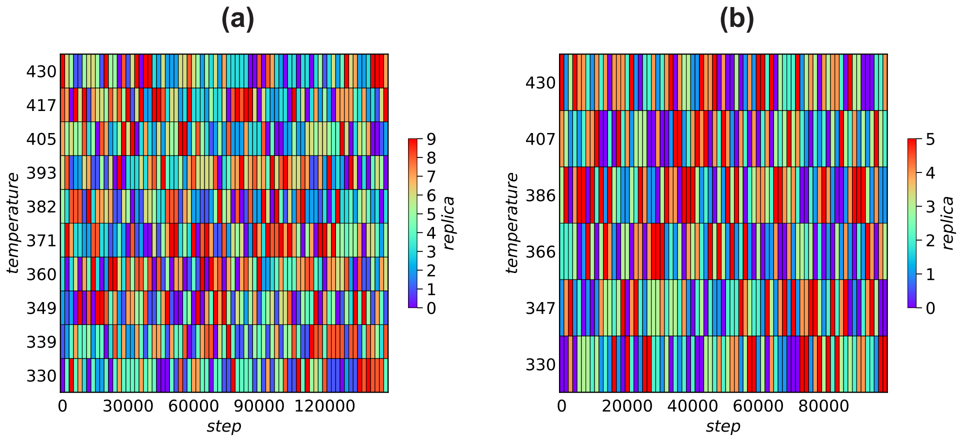

Fig. S2. Random walk of replicas over temperatures for a simulation trajectory taken from simulations of IL (a) binding to the DMPC bilayer and (b) in water. Scales on the right indicate the replica index, x-axes indicate the replica exchange step, and y-axes indicate temperatures sampled by replicas.

Simulation Convergence

Simulation equilibration was assessed by investigating turn content, $T(\tau)$, and the peptide center of mass along z, $z(\tau)$, as a function of simulation time τ, which represents the time collected at temperature $T_0 = 330$ K over REST trajectories. Turn was selected for convergence analysis because this is the dominant non-coil secondary structure type as shown in Fig. 2. Fig. S3a,c shows that $T(\tau)$ quickly converges within simulation time for both bilayer and water systems, and thus the peptide structure is largely insensitive to our definition of an equilibrated regime. Peptide $z(\tau)$ along the bilayer normal in Fig. S3b demonstrates a slower equilibration process, where the peptide is gradually adsorbed onto the bilayer and eventually reaches a stable position after $\tau \approx 250$ ns. These plots illustrate that our simulation systems have attained equilibration and can be used for structural analysis.

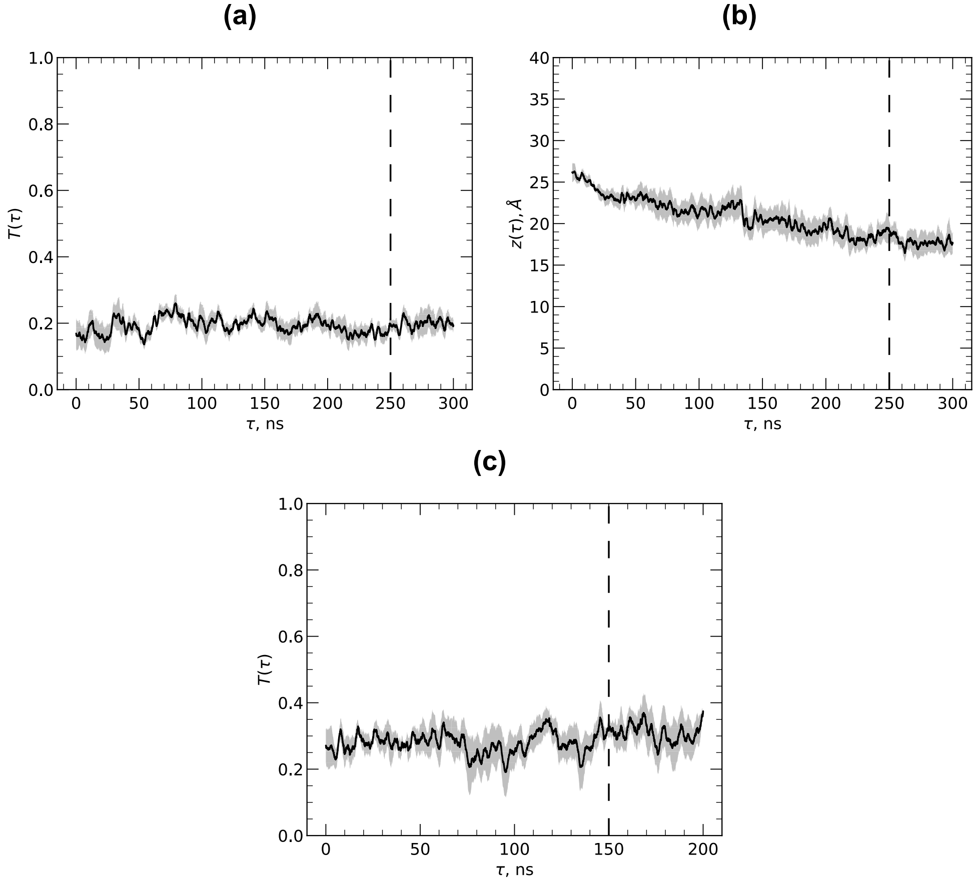

Fig. S3. Evaluation of simulation convergence along simulation time τ for (a) turn $T(\tau)$ in the bilayer system, (b) peptide center of mass distance from the bilayer midplane $z(\tau)$, and (c) $T(\tau)$ for the system in water. All quantities are averaged in windows of 2 ns. Shaded gray bands show standard errors. Vertical dashed lines indicate the points of convergence, which occur at 250 ns for bilayer simulations and 150 ns for water simulations.

Bilayer-Aware Clustering Alignment Protocol

To find dominant poses in our simulation trajectories, we performed a bilayer-aware clustering analysis. The Daura et al.[3] clustering algorithm requires the computation of pairwise distances between simulation structures. However, care must be taken when calculating pairwise distances to preserve peptide insertion along the bilayer normal (z axis). To solve for this issue, we implemented the Kabsch algorithm[4] in 2D along x and y, which permits translations and rotations along the bilayer plane during pairwise comparison but preserves z positions. This procedure is depicted in Fig. S4. Once the optimal alignment has been

performed, the root-mean-square deviation (RMSD) in 3D can be computed to quantify the similarity between structures. This procedure is designed to consider the difference in poses between peptide structures while retaining their insertion into the bilayer.

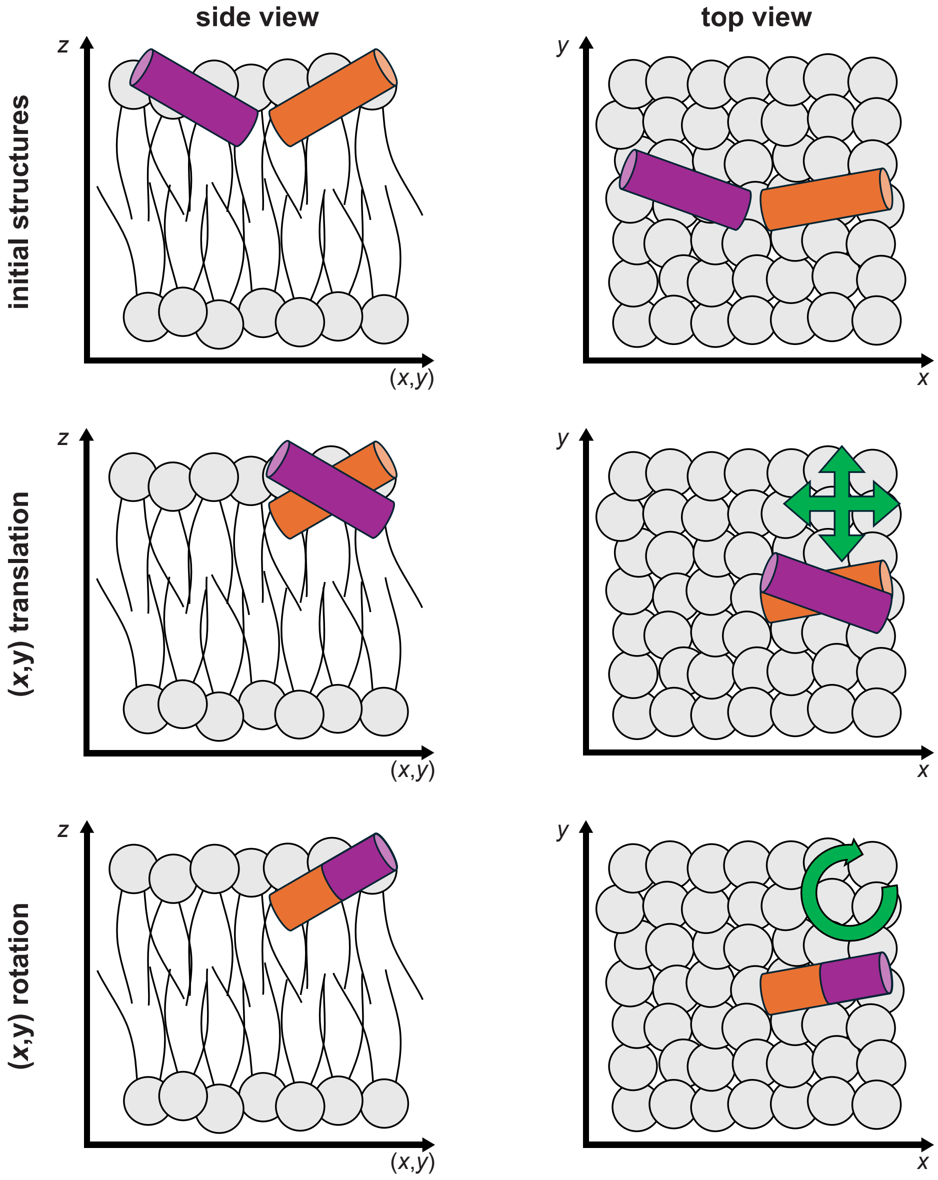

Fig. S4. Cartoon depicting the alignment protocol employed in our clustering analysis. Initial structures have a depth z along the bilayer normal and positions x and y in the bilayer plane. Two peptides are first translated to the same position on the (x,y) plane and then optimally rotated using a 2D implementation of the Kabsch algorithm.[4] Side and top views of the bilayer are provided for clarity. Two peptide structures are presented as purple and orange cylinders. Lipids are shown in gray. Green arrows indicate the steps involving translation or rotation within the bilayer plane.

Top Changes in Intrapeptide Contacts

To explore the changes in tertiary structure between water and bilayer environments, we computed $\Delta C(i,j) = \langle C_{bilayer}(i,j)\rangle - \langle C_{water}(i,j)\rangle$, showing the changes in contacts between residues i and j. The top 10 changes, all with $|\Delta C(i,j)| \geq 0.1$, are presented in Table S1. This table shows that all top changes in intrapeptide contacts involve a destabilization of these contacts in the bilayer environment with $\langle C_{bilayer}(i,j)\rangle < \langle C_{water}(i,j)\rangle$. Two of these top 10 (Pro3-Lys5 and Pro7-Trp9) are also featured in Table 1, which reports significant contacts with $\langle C(i,j)\rangle > 0.5$. Destabilization of these contacts may therefore act as important switches for determining the bilayer-bound or water conformational ensembles of IL.

Table S1. Top Changes in Intrapeptide Contacts

i-j	$\langle C_{bilayer}(i,j)\rangle$	$\langle C_{water}(i,j)\rangle$	$\Delta C(i,j)$
Pro3-Trp6	0.16 ± 0.03	0.39 ± 0.11	-0.23 ± 0.11
Leu2-Trp6	0.16 ± 0.03	0.37 ± 0.10	-0.21 ± 0.10
Pro3-Lys5	0.70 ± 0.06	0.88 ± 0.02	-0.18 ± 0.06
Lys5-Pro8	0.04 ± 0.01	0.23 ± 0.21	-0.18 ± 0.21
Pro7-Trp9	0.72 ± 0.05	0.87 ± 0.01	-0.15 ± 0.05
Trp6-Trp11	0.03 ± 0.01	0.16 ± 0.07	-0.13 ± 0.07
Trp4-Trp11	0.03 ± 0.01	0.15 ± 0.05	-0.12 ± 0.05
Leu2-Trp9	0.03 ± 0.00	0.14 ± 0.05	-0.12 ± 0.05
Pro3-Trp11	0.01 ± 0.00	0.12 ± 0.06	-0.11 ± 0.06
Leu2-Trp11	0.01 ± 0.00	0.11 ± 0.07	-0.10 ± 0.07

Inserted, Surface-Bound, and Unbound Probabilities

From the average distance $\langle z(i)\rangle$ of an amino acid i from the bilayer midplane, we computed the probability for i to be inserted P_i, surface bound P_s and unbound P_u (Table S2). For all amino acids, the insertion probability $P_i(i)$ is the most probable state. Moreover, $P_i(i) > 0.5$ for all i except Lys5. Comparing $P_s(i)$ and $P_u(i)$, the N-terminal amino acids Ile1-Trp8 favor an unbound state with $P_u(i) > P_s(i)$ whereas when uninserted C-terminal amino acids Trp9-Arg13 favor the surface bound state with $P_s(i) > P_u(i)$.

Table S2. Probability of Inserted, Surface Bound, and Unbound Amino Acids.

i	$P_i(i)$	$P_s(i)$	$P_u(i)$
1	0.59 ± 0.04	0.10 ± 0.02	0.31 ± 0.05
2	0.57 ± 0.08	0.10 ± 0.06	0.34 ± 0.05
3	0.55 ± 0.08	0.12 ± 0.07	0.33 ± 0.05
4	0.52 ± 0.04	0.15 ± 0.04	0.33 ± 0.04
5	0.45 ± 0.07	0.23 ± 0.06	0.32 ± 0.07
6	0.55 ± 0.09	0.19 ± 0.06	0.26 ± 0.05
7	0.53 ± 0.08	0.21 ± 0.05	0.25 ± 0.06
8	0.63 ± 0.09	0.17 ± 0.04	0.20 ± 0.07
9	0.66 ± 0.09	0.17 ± 0.04	0.17 ± 0.05
10	0.62 ± 0.08	0.21 ± 0.04	0.18 ± 0.07
11	0.64 ± 0.09	0.24 ± 0.05	0.12 ± 0.04
12	0.57 ± 0.07	0.36 ± 0.05	0.07 ± 0.03
13	0.54 ± 0.03	0.38 ± 0.04	0.08 ± 0.02

Green, yellow, and pink highlights show the 1st, 2nd, and 3rd most probable states.

Distribution of Tryptophan Residues

Experiments of IL and POPC large unilamellar vesicles have shown that Trp is ∼11 Å from the bilayer midplane.[5] In Fig. S5, we show the probability distribution $P_{Trp}(z)$ of Trp side chain distances z from the bilayer midplane. The peak in this distribution at $z = 10$ Å is in good agreement with experiments. Our simulations also show a less pronounced peak at $z = 23$ Å, which corresponds to Trp surface bound to the DMPC bilayer.

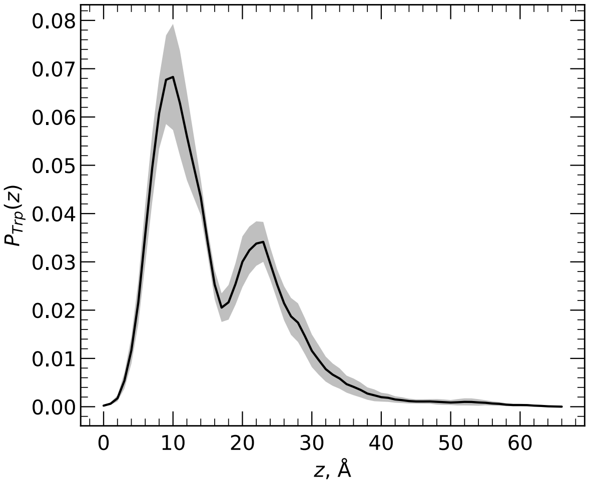

Fig. S5. Probability distribution $P_{Trp}(z)$ of Trp residue side chain distances z from the bilayer midplane. The shaded gray band shows the standard error.

Binding Profile of Clusters

To supplement our bilayer-aware clustering analysis, we computed the average distance $\langle z(i;c)\rangle$ of each amino acid i center of mass from the bilayer midplane for each populated cluster c (Fig. S6). This figure shows that clusters sample a gradient of inserted, surface bound, and unbound amino acid states. In all clusters, C-terminal amino acids Arg12 and Arg13 reside at approximately z_P, indicating that this positioning is a hallmark of IL binding to the DMPC bilayer.

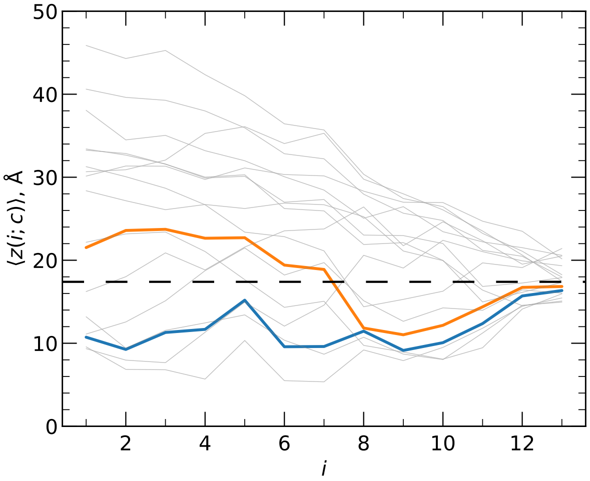

Fig. S6. Distance $\langle z(i;c)\rangle$ of each amino acid i center of mass from the bilayer midplane for populated clusters c. The profiles for top clusters 1 and 2 are in blue and orange, respectively. Thin gray lines show the other 15 clusters. The dashed horizontal line represents z_P.

References

(1) Smith, A. K.; Lockhart, C.; Klimov, D. K. Does Replica Exchange with Solute Tempering Efficiently Sample Aβ Peptide Conformational Ensembles? *J. Chem. Theory Comput.* **2016**, *12*, 5201–5214.

(2) Han, M.; Hansmann, U. H. E. Replica Exchange Molecular Dynamics of the Thermodynamics of Fibril Growth of Alzheimer's Disease Aβ_{42} Peptide. *J. Chem. Phys.* **2011**, *135*, 065101.

(3) Daura, X.; Gademann, K.; Jaun, B.; Seebach, D.; van Gunsteren, W. F.; Mark, A. E. Protein Folding: When Simulation Meets Experiment. *Angew. Chem. Int. Ed.* **1999**, *38*, 236–240.

(4) Kabsch, W. A Solution for the Best Rotation to Relate Two Sets of Vectors. *Acta Cryst.* **1976**, *A32*, 922–923.

(5) Ladokhin, A. S.; Selsted, M. E.; White, S. H. Bilayer Interactions of Indolicidin, a Small Antimicrobial Peptide Rich in Tryptophan, Proline, and Basic Amino Acids. *Biophys. J.* **1997**, *72*, 794–805.
